# A meta-analysis of financial self-control strategies: Comparing empirical findings with online media and lay person perspectives on what helps individuals curb spending and start saving

**DOI:** 10.1371/journal.pone.0253938

**Published:** 2021-07-08

**Authors:** Mariya Davydenko, Marta Kolbuszewska, Johanna Peetz

**Affiliations:** Carleton University, Ottawa, ON, Canada; Xiamen University - Malaysia Campus, MALAYSIA

## Abstract

Self-control can be assisted by using self-control strategies rather than relying solely on willpower to resist tempting situations and to make more goal-consistent decisions. To understand how self-control strategies can aid financial goals, we conducted a meta-analysis (Study 1) to aggregate the latest research on self-control strategies in the financial domain and to estimate their overall effectiveness for saving and spending outcomes. Across 29 studies and 12 different self-control strategies, strategies reduced spending and increased saving significantly with a medium effect size (*d* = 0.57). Proactive and reactive strategies were equally effective. We next examined whether these strategies studied in the academic literature were present in a media sample of websites (*N* = 104 websites with 852 strategies) and in individuals’ personal experiences (*N* = 939 participants who listed 830 strategies). About half the strategies identified in the meta-analysis were present in the media sample and about half were listed by lay participants as strategies they personally use. In sum, this paper provides a comprehensive overview of the self-control strategies that have been studied in the empirical literature to date and of the strategies promoted in the media and used in daily life, identifying gaps between these perspectives.

## Introduction

Any goal pursuit is fraught with self-control dilemmas, where the individual must choose between indulging in temptation right now versus resisting temptation and pursuing the goal consistent choice. For instance, when aiming to limit one’s spending, daily temptations (e.g., to buy a new gadget, a favourite drink, or book a vacation) must be resisted to act in line with the goal. Often, temptations prevail and individuals fall short of their goal. For example, 44% of American adults self-report that their retirement savings are not on track and 26% have no retirement savings or pension whatsoever [1]. In America, 74% of people have credit card debt [2], impulse purchases account for as much as 60% of all purchases [3], and 44.4% of bankruptcies were related to spending or living beyond one’s means [4].

What are some ways to bring people’s actions in line with their financial goals? One way to increase goal-consistent choices and promote self-control in tempting situations is the employment of self-control strategies [5]. Self-control strategies are a form of self-management that can make goal-consistent choices more likely while reducing the need for willpower to resist temptations. For example, people tend to spend less money if they make the purchase with cash rather than a card [6–8]. Therefore, keeping cash on hand rather than a credit card may be a helpful strategy to deter oneself from spending in a tempting situation. Such strategies and tactics are specific, actionable ways to increase one’s chances of making goal-consistent choices. In the present research we examine self-control strategies for financial goals from three unique perspectives: (1) strategies empirically studied by researchers, (2) strategies recommended in the online media, and (3) strategies described by a sample of lay individuals. Understanding the match or mis-match of self-control strategies across these three perspectives can provide future directions for self-control theory development and experimentation, as well as highlight the state of knowledge translation between researchers and the public.

### Self-control

Self-control is characterized by a conflict between two opposing forces: between the myopic doer and the far-sighted planner [9], between desire and willpower [10], between wants and shoulds [11], or between immediate gratification and enduring goals [12]. Common across these conceptualizations of self-control is the struggle between a desire and a goal. Temptation occurs when the desire is at odds with the person’s goals [25]. An abundant amount of research on trait self-control suggests the ability to control one’s impulses has wide-reaching benefits in terms of eating behaviour, weight control, academic and work performance, well-being, and prosocial behaviour [13].

Is self-control relevant to financial decisions? Individuals high in trait self-control have a greater propensity to plan and make budgets [14], engage in less impulsive buying [15], have fewer credit cards and are less likely to have a revolving monthly credit card balance [16], and are more likely to contribute to their retirement savings [17]. In-the-moment depletion of self-control resources has been shown to increase impulsive buying [18].

### Self-control strategies

Controlling one’s impulses and instead making decisions that adhere to one’s goals is not easy. Self-control conflicts are considered more difficult, tiring, and effortful than situations without a self-control element [19]. One way to resist temptations is to exert willpower, suppressing the tempting impulse. However, exerting willpower is effortful [20, 21] and cognitively costly [22]. Another way to resist temptations and to act in line with goals is the use of self-control strategies.

Self-control strategies are “little tricks we play on ourselves to make us do the things we ought to do or to keep us from the things we ought to foreswear” [23 p. 290]. Strategies can include everything an individual does or thinks either before or during a tempting situation that helps them resolve self-control conflicts between competing short-term gains and long-term benefits. Self-control strategies can ease the subjective experience of exerting willpower (i.e., make the temptation less tempting), suppress the goal-inconsistent choice (i.e. make choosing the tempting option more difficult), or promote the long-term goal (i.e., make choosing the goal-consistent option easier). Such strategies are specific, actionable ways to increase a person’s chances of making goal-consistent choices–like self-imposed “nudges” [24].

Self-control strategies can be conceptually organized by the focus of the strategy (i.e., self-control versus willpower strategies; [10]), the process of the strategy (i.e., situational versus intrapsychic strategies; [5]), and the timing of the strategy (i.e., preventative versus interventive strategies; [25]). A major distinction is the organization of strategies into those that are employed *proactively* before encountering a spending temptation (e.g., ‘proactive strategies’ [26], ‘preventive strategies’[25], ‘situational strategies’[5]), and those used *reactively* after encountering a spending temptation (e.g., ‘reactive strategies’ [26], ‘interventive strategies’ [25], ‘intrapsychic strategies’[5]). For example, *proactive self-control strategies* focus on behaviors and cognitions people can do in anticipation of a tempting situation (e.g., avoid walking by a cake shop when on a diet), and *reactive self-control strategies* focus on the behaviors and cognitions people can do once they are experiencing the tempting situation (e.g., distract yourself by drinking water when tempted by doughnuts at the office).

When people anticipate a temptation in the future, their ability to make goal-consistent decisions improves [27, 28]. According to models of self-control, proactive strategies are superior to reactive strategies because they are forward thinking, are deployed earlier in the self-control cycle, and can trigger further strategies at later stages of the self-control cycle [5]. In a study on self-control in an academic context, students assigned to employ a proactive self-control strategy made greater progress on their academic goal than students instructed to use will power or received no instruction [29]. Similarly, in a study across different types of goal, participants instructed to use a proactive self-control strategy (i.e., anticipate obstacles and plan ways to overcome the obstacles) made greater goal progress than those who were given no instructions [27]. One downside of proactive strategies, however, is that they require foresight, planning, and control over the situation to be effective. A recent experience sampling study across different types of goals showed that people successfully used proactive strategies in only 9% of their self-control conflicts [30]. Reactive strategies can be helpful for unexpected, unprepared for, or unavoidable temptation and include a variety of behavioral and cognitive techniques, such as distraction and reappraisal, to reduce the strength of the temptation [5, 25]. Reactive strategies have also been shown to be effective [31]. Recent research using experience sampling methods, asked people to report on unpleasant activities that included a self-control element, as well as what spontaneous reactive strategies they used to boost their self-control and to perform the activity [31]. The researchers found that during the unpleasant activity, focusing on the positive outcomes of the activity, thinking about how the activity is almost completed, regulating one’s emotions, and monitoring progress, were the most effective reactive strategies for increasing self-control. In the domain of spending, both proactive and reactive self-control strategies may be effective. We examine the prevalence of these strategies in the academic literature on financial saving and spending behaviors, in a sample of online media recommendations of strategies, and in a sample of lay person’s self-reported strategy use.

### Integrating perspectives on self-control strategies

Outside of academic literature, self-control for financial goals is a popular topic. A quick Google search returns 648,000,000 results for “spend less money and self-control” and 778,000,000 results for “save more money and self-control”. People are interested in learning about how to use self-control for financial goals and a variety of people have something to say about it. From financial advisors to journalists to bloggers, people dispense advice online for how to boost self-control to save more or spend less money. Although these recommendations are not usually based on empirical findings, this is the advice that most lay people find when looking for help with their self-control. Therefore, the purpose of this research is to examine not only the academic literature, but also the online media and lay people’s reports on self-control strategies. These multiple perspectives allowed us to investigate how many of the self-control strategies studied in the academic literature are circulated in the online media and are known by the public, as well as to identify gaps between the perspectives.

### Present research

The purpose of the present research was twofold. First, to create a rigorous collection of empirically studied proactive and reactive financial self-control strategies, and second, to compare academic, media and lay perspectives on proactive and reactive financial self-control strategies. To address the first purpose, we identified empirically studied self-control strategies for financial decisions through a formal meta-analysis, including research from psychology, marketing, and economics. To our knowledge, this is the first meta-analysis on self-control strategy use in the financial domain. The objectives of the meta-analysis were to collate financial self-control strategies, to measure of the overall effectiveness of self-control strategies, and to examine whether effect sizes for proactive and reactive self-control strategies differed. To address the second purpose, we examined self-control strategies promoted in the online media, again collating financial self-control strategies and examining the relative prevalence of proactive versus reactive strategies. Finally, we examined the self-control strategies lay individuals described using in their daily life. The online media and lay perspective may reveal financial self-control strategies that have yet to be empirically tested but are commonly used. The studies examining the online media and lay perspectives were preliminary investigations of self-control strategies outside the academic literature and are based on convenience samples.

## Study 1: A meta-analysis of financial self-control strategies in the academic literature

We conducted a formal meta-analysis of the existing literature on empirically tested self-control strategies in the financial domain. We collected experimental studies based on adult samples that compared a self-control strategy to a control treatment and measured saving or spending as an outcome. This approach aimed to provide an overview of the current state of the research but also provided an initial estimate of the effectiveness of financial self-control strategies in an experimental context. We also examined whether strategy effectiveness differed by type of strategy (proactive vs. reactive strategies).

### Method

#### Identification process

**Fig 1:**
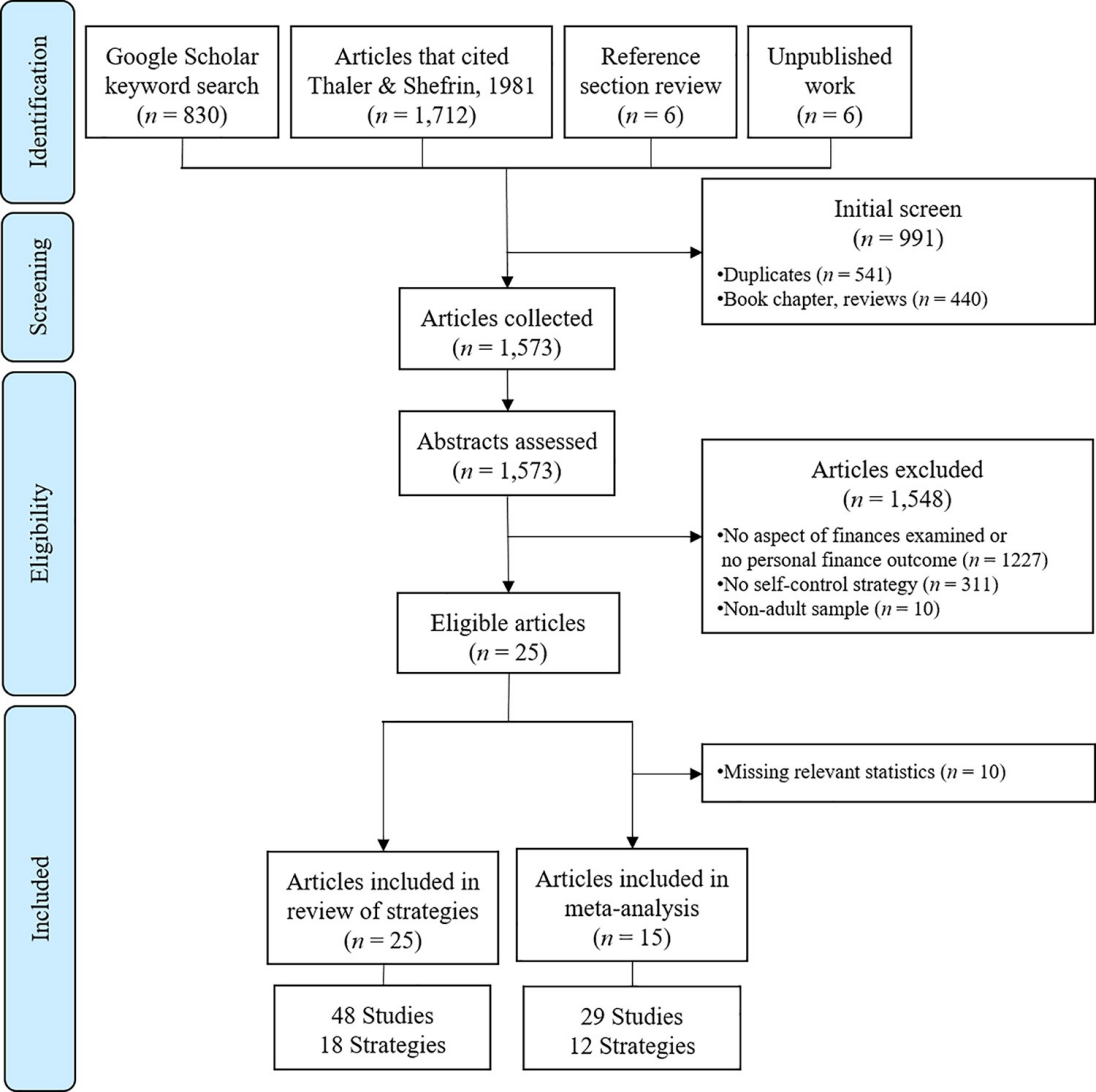
Flow diagram of screening process.

In a first step of the literature search, we searched relevant databases, including PsycInfo, SAGE Journals Online, Wiley Online Journals, and ProQuest via the meta-search engine Google Scholar, for articles of any type (i.e., published, dissertation, grey papers, pre-prints) that matched these search terms: “allintitle: “saving decisions””, “allintitle: “saving behaviour””, “allintitle: “personal saving””, “allintitle: “spending decisions””, “allintitle: “spending behavior””, “allintitle: “personal spending””. There were no date constraints on the search, but the search parameters were constrained to articles in English. In a second step, we included papers which cited a hallmark paper on financial self-control [9]. This seminal paper brought attention to the concept of self-control in the economic literature and introduced more research from the economics discipline into our meta-analysis. In a third step, we screened the reference sections of articles identified in the search. In a fourth step, we put out a call for unpublished or in-press papers on several listserv platforms (i.e., Society of Personality and Social Psychology, European Association for Social Psychology, Society for Judgment and Decision Making). The search for relevant research was initially conducted in July 2018 and updated in July 2020. Fig 1 depicts the flow of the search and screening process and the number of articles found in each of the steps of the article identification process. A total of 1,573 unique texts were identified.

#### Screening and eligibility process

Next, the abstracts of all remaining articles were evaluated for eligibility on several criteria. When coding for eligibility, coding stopped once an article was deemed ineligible according to any one of the criteria. First, an article was eligible if it examined individual-level spending or saving. We excluded those articles that did not assess spending or saving outcomes (e.g., research on stocks, dividends, non-finance related outcomes) and those that did not assess *individual-level* spending outcomes (e.g., research on spending or saving at the household, organizational, or country level). Second, an article was eligible if it examined self-control strategies as a predictor variable. We excluded articles treating the use of self-control strategies as an outcome variable (e.g., relationship between individual differences and strategy use) and excluded articles that examined ways to reduce spending or increase saving outside the individual’s control (e.g., defaults set up by organizations not individuals). Third, an article was eligible if it examined adult samples. We excluded studies conducted with children or adolescents. There were no other inclusion or exclusion criteria. The first author screened the abstracts of all 1,573 articles for eligibility on these criteria. A second coder screened a random sample of 200 abstracts. Interrater reliability for article inclusion was strong (98% agreement) and significant, κ = .84 (95% CI [.68, .99]), *p* < .001 [32, 33].

A total of 25 articles were deemed eligible. These articles outlined 18 distinct financial self-control strategies. Half of these strategies were proactive strategies that focused on what people can do before they encounter a tempting situation: using a retirement savings projection plan [34], tracking weekly saving deposits [35], automatizing savings [36, 37], using a savings account with no early withdrawals [38], setting specific saving goals [39, 40], keeping money in an account rather than in cash [41], making the saving goal personally meaningful [42], keeping budgets for shopping trips [43, 44], and planning purchases with a shopping list [45]. The other half were reactive strategies that focused on what people can do during a tempting situation: imagining one’s future self [46, 47], considering why you are pursuing the goal [48], keeping cash in large denomination bills [49] or in bills rather than coins [50], paying with cash as opposed to cards [6–8], organizing expenses into categories [51], tracking expenses via text messages [52], avoiding financing options by paying now rather than later [53], remembering your account balance before thinking about a future purchase [51], and anticipating future regret over purchases [54]. Using coupons was the only self-control strategy that *in*creased spending [55].

However, for 10 of these 25 articles the necessary meta-analysis statistics were not reported in the article (and could not be obtained by contacting the authors). To be included in the meta-analysis, the articles needed to report at least one of the appropriate statistics needed to compute the effect size (i.e., *M* and *SD* or *t*, or *F*), as well as the total sample size and the sample size per condition. From these reported statistics, we computed Cohen’s *d* for each study using online calculators [56] based on the difference in means between the strategy and control group [57]. In the case of repeated measure study designs, only the first post-test measurement was used for analysis to allow for meaningful comparison with independent-group study designs that reported only one measurement after the strategy intervention. By including only one effect size from each study, we prevented having the same participant represented in more than one effect size estimate. Thus, the meta-analysis is based on 15 articles with a total of 29 studies testing 12 unique self-control strategies, with 29 effect sizes (see Table 1). Next, these 29 studies were coded for study design, sample, intervention, and outcome characteristics, as well as within study bias coding. The data for the meta-analysis and for Study 2 and 3 are available on the Open Science Framework (OSF) at https://osf.io/vpwfd/?view_only=ff460390e6f040f997131d907c75b4ba.

**Table 1:** Studies included in the meta-analysis, in order of effect size of the financial self-control strategies.

	Study	Strategy	Strategy condition (vs. control condition)
1	Raghubir & Srivastava, 2009 (s1a)	Keep cash in large denomination	Pay with 1 x $1 bill (vs. 4 x $0.25 coins)
2	Fajnzylber & Reyes, 2015	Use a savings projection plan	See personalized retirement saving projection statement (vs. not)
3	Davydenko & Peetz, 2020 (s1)	Write a shopping list	Write a shopping list (vs. not)
4	Rudzinska-Wojciechowska, 2017 (s2)	Think about reason for goal	Adopt abstract mindset (vs. concrete mindset)
5	Prelec & Simester, 2001 (s2)	Pay cash only	Pay with cash (vs. credit card)
6	Akbaş et al., 2016	Track account	Track weekly saving deposits (vs. weekly text message reminders)
7	Davydenko & Peetz, 2020 (s2)	Write a shopping list	Write a shopping list (vs. not)
8	Beshears et al., 2011 (s2)	No early withdrawals	Use a savings account with no early withdrawal (vs. early withdrawal penalty)
9	Sheehan & Van Ittersum, 2018 (s4)	Have a budget	Shopping with a budget (vs. without a budget)
10	Raghubir & Srivastava, 2009 (s1c)	Keep cash in large denomination	Pay with 1 large bill (vs. 5 smaller denomination bills)
11	Hernández Escuer et al., 2014	Track account	Receive text messages listing each expenditure (vs. not)
12	Helion & Gilovich, 2014 (s2)	Pay cash only	Pay with cash (vs. gift card)
13	Raghubir & Srivastava, 2009 (s1b)	Keep cash in large denomination	Pay with 1 x $5 bill (vs. 5 x $1 bill)
14	Hershfield et al., 2011 (s1)	Imagine future self	See photo of self aged to 70 years old (vs. unaltered photo)
15	Beshears et al., 2011 (s1)	No early withdrawals	Use a savings account with no early withdrawal (vs. early withdrawal penalty)
16	Raghubir & Srivastava, 2008 (s3)	Pay cash only	Pay with cash (vs. gift card)
17	Raghubir & Srivastava, 2008 (s2)	Pay cash only	Pay with cash (vs. credit card)
18	Tam & Dholakia, 2011 (s1)	Make goal specific	Set savings goal for a specific future month (vs. next month)
19	Raghubir & Srivastava, 2008 (s4)	Pay cash only	Pay with cash (vs. gift card)
20	Prelec & Simester, 2001 (s1)	Pay cash only	Pay with cash (vs. credit card)
21	Tam & Dholakia, 2011 (s3)	Make goal specific	Set savings goal for a specific future month (vs. next month)
22	Rudzinska-Wojciechowska, 2017 (s3)	Think about reason for goal	Think about why saving money (vs. think about how to save money)
23	Hershfield et al., 2011 (s3a)	Imagine future self	See aged avatar of current self (vs. unaltered avatar of current self)
24	Tessari et al., 2011 (s3)	Keep cash in bills	Pay with fake currency banknotes (vs. fake currency coins)
25	Tessari et al., 2011 (s1a)	Keep cash in bills	Pay with fake €1 banknotes (vs. fake €1 coins)
26	Tessari et al., 2011 (s1b)	Keep cash in bills	Pay with fake €1 banknotes (vs. fake €1 coins)
27	Tessari et al., 2011 (s2)	Keep cash in bills	Pay with real $1 banknotes (vs. real $1 coins)
28	Somville & Vandewalle, 2018	Make money hard to access	Keep money in a bank account (vs. keep it in cash)
29	Tam & Dholakia, 2011 (s5)	Make goal specific	Set savings goal for a specific future month (vs. future quarter)

### Results

#### Descriptives of the studies and samples

The majority of the studies were published articles in peer-reviewed journals (75.9%), conducted in the U.S. (53.6%), and recruited university student samples (65.5%). Most studies (77.8%) were conducted in person (in-lab: *n* = 18, in-person outside of lab: *n* = 5, online: *n* = 6). Across all studies, a total of 12,316 participants were recruited (*M*_age_ = 26.6 and 65.2% of studies had a roughly equal gender distribution). The sample size of studies varied widely, from 24 to 8,940 participants. The year of publication ranged from 2001 to 2020 and 65.5% (*n* = 19) of the studies were published before 2012 –before the replication crisis in social psychology [58].

#### Overall effect size of strategy use

According to Cohen [59], *d* = .20 is a small effect size, *d* = .50 is a medium effect size, and *d* ≥ .80 is a large effect size. All studies were coded such that the effect reported was in the direction of lower spending or higher saving, respectively. In all analyses reported below we report results obtained after a random-effects model was applied.

**Fig 2:**
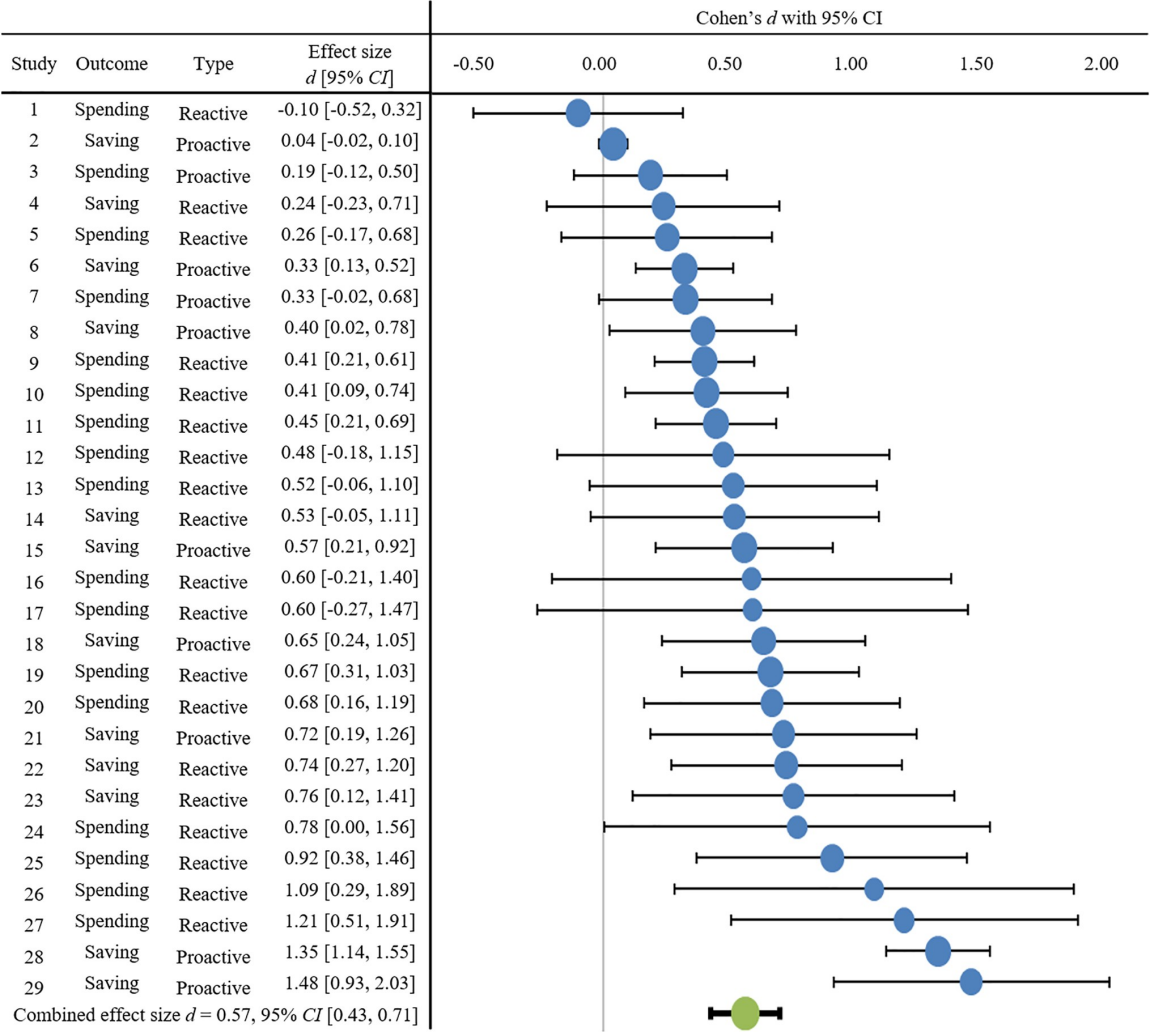
Forest plot of strategy use effect size by study. Each line represents one observation. The position of the bubble depicts the effect size. The size of the bubble represents the weight of that individual study on the overall average effect size, whereby studies with larger sample sizes are weighed more and are represented with larger bubbles. The green bubble represents the weighted combined effect size. The bars represent the 95% confidence interval. Outcome refers to whether the purpose of the strategy is to reduce spending (Spend) or increase saving (Save). Type refers to whether the strategy is proactive (PRO) or reactive (RE). Refer to Table 1 for strategy details.

The average effect size of financial self-control strategies was 0.57 with a 95% confidence interval from 0.43 to 0.71 (*Z* = 8.38, *p* < .001). In other words, on average, instructing participants to employ a financial self-control strategy successfully reduced spending or increased saving with a medium effect size [59]. A forest plot of effect sizes by study is depicted in Fig 2. Examining the forest plot revealed there were no effect size outliers. However, there was one study (study #2; [34]) that was a sample size outlier and due to its large sample size (*n* = 8940) was weighted most heavily in the calculation of the combined effect size. Sensitivity analyses showed that excluding this study from the meta-analysis did not change the combined effect size (*d* = 0.59, 95% CI [0.45, 0.73], *Z* = 8.75, *p* < .001). Of the 29 studies, 12 had effect size confidence intervals that included zero.

#### Homogeneity of effect sizes

Next, we examined the variability in effect sizes. The homogeneity test of the overall effect size was significant (*Q* = 241.68, *df* = 28, *p* < .001), indicating that there was significant heterogeneity in effect sizes and that the observed variation in study effect sizes was larger than would be expected from mere sampling error. The proportion of total variation due to heterogeneity between studies was large (*I*^2^ = 88.4%; [60]), suggesting the variability in effect sizes is due to heterogeneity rather than sampling error. This heterogeneity suggests other factors need to be accounted for in order to explain the variability of the true effect sizes and it supports the random-effects model approach.

#### Moderator analysis

We examined whether effect sizes diffed for studies testing proactive strategies and those testing reactive strategies. Across all included studies, 11 (37.9%) examined proactive strategies, and 18 (62.1%) examined reactive strategies. A meta-regression revealed that strategy type was not a significant predictor of effect size (β = 0.02, *B* = 0.13, *SE* = 0.18, *F*(1, 27) = 0.01, *p* = .930). Strategy type explained only 0.03% of the variance in the overall effect size. The combined effect size for proactive strategies (*d* = 0.56, 95% CI [0.30, 0.83]) was not larger than the combined effect size for reactive strategies (*d* = 0.58, 95% CI [0.43, 0.72]).

### Risk of bias

*Within study bias*. As with any meta-analysis, the conclusions drawn from this meta-analysis are restricted to the methodological rigor of the included studies. We coded individual studies included in this meta-analysis for risk of bias, specifically for selection bias (random sequence generation and allocation concealment), performance bias, detection bias, attrition bias, and selective reporting bias. Random sequence generation refers to whether participants were assigned to conditions using non-random (high risk) or random (low risk) approaches. Allocation concealment refers to whether participants and researchers could foresee (high risk) or could not foresee (low risk) into which condition an upcoming participant would be assigned. Performance bias refers to whether participants and researchers knew (high risk) or did not know (low risk) which participants received which intervention. Detection bias refers to whether the participants knew (high risk) or did not know (low risk) the study’s outcome of interest. Attrition bias refers to whether conditions differed (high risk) or did not differ (low risk) in the amount, nature, or handling of incomplete outcome data. Selective reporting bias refers to whether reported findings differed (high risk) or did not differ (low risk) from unreported findings. Two coders (i.e., the first author and an additional rater) rated each study as high risk, low risk, or unclear risk of bias (see Table 8.5.c in [61]) for risk assessment tool used). Overall, there was moderate to strong agreement (κ range = .65 to 1.00; [32]) between the raters with 93.1% to 100% consistent ratings: 96.6% agreement for selection bias in random sequence generation, κ = .842, *SE* = .154; 96.6% agreement for selection bias in allocation concealment, κ = .651, *SE* = .322; 100% agreement for performance bias, κ = 1.00; 96.6% agreement for detection bias, κ = .655, *SE* = .317; 93.1% agreement for attrition bias, κ = .847, *SE* = .104; and 96.6% agreement for selective reporting bias, κ = .838, *SE* = .157. Inconsistencies were resolved by the first author.

The majority of studies included in the meta-analysis were judged to be low in risk for selection bias (i.e., groups were randomly assigned), performance bias (i.e., participants and researchers did not know which participants were in which group), and detection bias (i.e., participants did not know the main outcome). Judgments of attrition bias and selective reporting bias were mostly coded as “unclear risk” because of missing information, such as no reasons for missing data were stated or the study protocol was not available to permit a judgement of either low or high risk. We expected it would be difficult to assess risk of selective reporting bias [61], considering most of the studies were conducted before open science practices were more widely accepted. See Table 2 for the breakdown by judgments of bias. Due to the lack of heterogeneity in risk of bias ratings for the included studies, we did not examine within study bias as a moderator of overall effect size.

**Table 2:** Frequency table of within study risk of bias in meta-analysis.

Type of bias	Risk of bias (*k* = 29)			Inter-rater reliability
	Low risk *n* (%)	High risk *n* (%)	Unclear risk *n* (%)	
Selection bias: random sequence generation	26 (89.7)	1 (3.4)	2 (6.9)	κ = .842, *SE* = .154
Selection bias: allocation concealment	27 (93.1)	0	2 (6.9)	κ = .651, *SE* = .322
Performance bias	29 (100)	0	0	κ = 1.00
Detection bias	28 (96.6)	1 (3.4)	0	κ = .655, *SE* = .317
Attrition bias	9 (31.0)	1 (3.4)	19 (65.5)	κ = .847, *SE* = .104
Selective reporting bias	3 (10.3)	0	26 (89.7)	κ = .838, *SE* = .157

**Fig 3:**
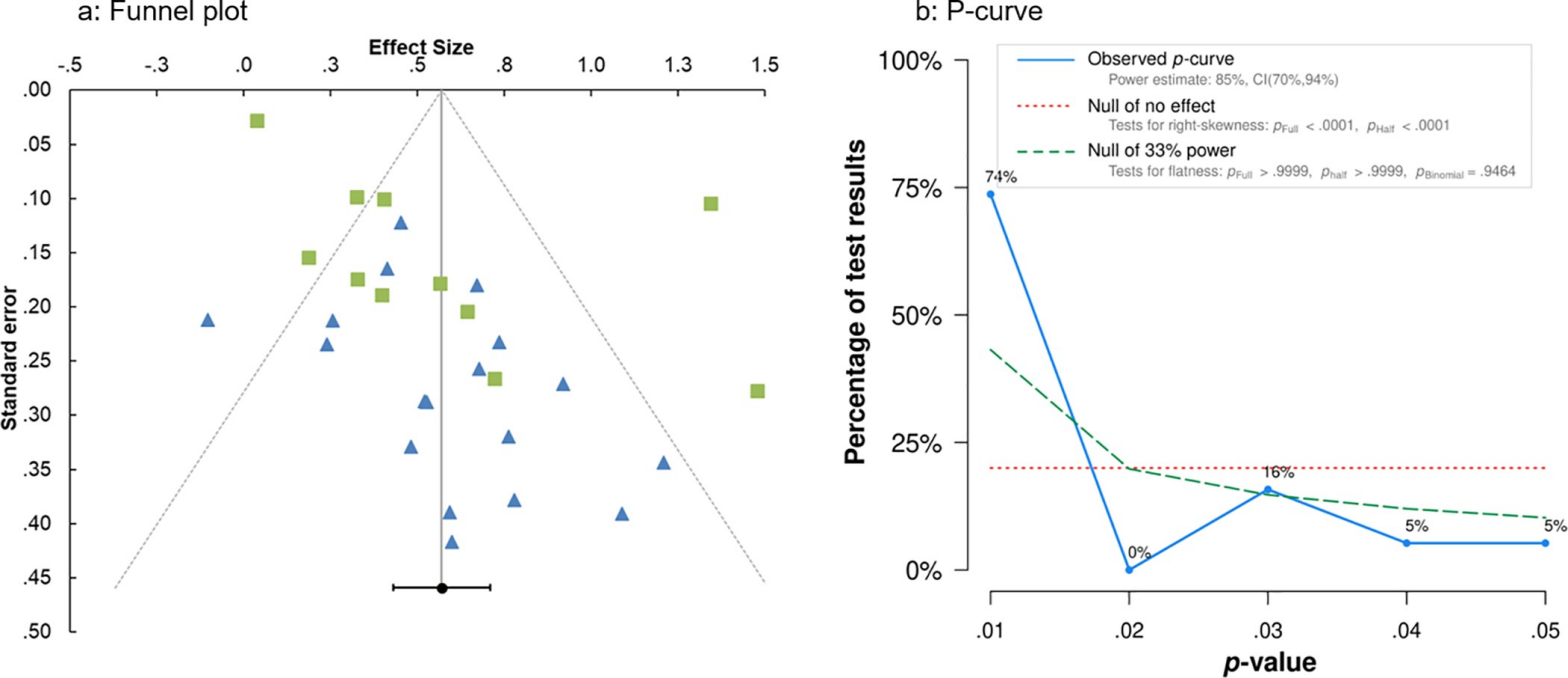
Presence of publication bias and p-hacking in meta-analysis. Fig 3A shows the funnel plot of individual effect sizes by strategy type. Blue triangles represent reactive strategies and green squares represent proactive strategies. The vertical red line represents the overall effect size (*d* = 0.57) with the red diagonal lines representing the 95% confidence interval. Fig 3B shows the p-curve distribution of p-values from meta-analysis.

*Publication bias and p-hacking*. We examined whether the effect sizes included in the meta-analysis were biased due to a lack of publication of studies with small or nonsignificant effect sizes using a funnel plot. A funnel plot (Fig 3) of the studies revealed signs of publication bias; specifically there were more published studies with effect sizes greater than the overall effect size (i.e., the right portion of the funnel plot) than studies with effect sizes less than the overall effect size (i.e., the left portion of the funnel plot). Such a distribution suggests that studies which found small or null effects are missing from the published literature. Studies that examined reactive strategies had generally larger standard errors and reported larger effect sizes (Fig 3A); this is indicative of the tendency for smaller studies to find larger effect sizes [62] and might suggest greater publication bias in studies testing reactive strategies than proactive strategies. However, it is important to note that visual examination of a funnel plot is too subjective to provide conclusive evidence [63]. I also conducted an Egger’s regression to examine whether there is a significant linear relationship between the effect sizes and the standard errors which would suggest publication bias [64]. The Egger’s regression was significant (*B* = -0.92, *SE* = 0.48, *t*(23) = 3.12, *p* = .005), supporting the presence of publication bias.

We also conducted Rosenthal’s and Orwin’s *Fail-safe N* tests to further explore the potential impact of publication bias on the meta-analysis findings. Rosenthal’s *Fail-safe N* [63] suggests 2,385 studies with null effects are needed before the overall effect size becomes not significant. Orwin’s *Fail-safe N* [65] suggests 54 studies with a null effect are needed to lower the overall effect size found from a medium (*d* = 0.57) to below a small (*d* = 0.20) effect [59]. These *Fail-safe N* tests should be interpreted with caution because they are a measure of the tolerance of the overall effect size to additional null results rather than a measure of the file drawer problem (i.e., how many null results are hidden in the literature).

We also examined whether the studies included in the meta-analysis showed potential *p*-hacking [66–68] through a *p*-curve (Fig 3). From the 29 studies in the meta-analysis, the *p*-curve included 19 statistically significant results at *p* < .05, of which 16 were *p* < .025. The *p*-curve showed there was a significant positive skew (binomial test *p* = .002) with more low than high *p*-values, suggesting evidential value. The estimated statistical power of tests included in the *p*-curve is 85% with a 90% confidence interval of 70% to 94% power. Therefore, although the funnel plot and the Egger’s regression suggested that there was publication bias among the studies in the meta-analysis, the *p*-curve suggested that the published findings have evidential value.

### Discussion

As the first systematic review of literature on financial self-control strategies, this study aggregated 18 self-control strategies that have been experimentally tested. A formal meta-analysis of a subset of these strategies (i.e., those with available effect sizes) showed that in these experimental tests, self-control strategies effectively reduced spending or increased saving (89.7% of studies found at least a small effect size, *d* ≥ 0.20; 58.6% of studies found at least a medium effect size, *d* ≥ 0.50; [59]). Given the presence of publication bias, it is possible that other strategies have been tested experimentally but shown no effect, or that the overall effect size of self-control strategies on financial behavior is actually lower than this published research suggests [69, 70]. Across the reviewed papers, however, this study shows that there are effective financial self-control strategies people may use to increase goal-adherence.

The type of these empirically studied strategies was evenly split between reactive (vs. proactive) strategies. Although the meta-analysis showed no evidence for moderation by type of strategy or by financial outcome, it should be noted that these results should be interpreted with caution. Strategies coded as reactive in the experimental context might also be employed as proactive strategy outside the lab. For example, if the study compared paying with cash or credit card for a purchase, “paying cash” was coded as reactive strategy because it was used during the experimental spending situation [6]. However, if one were to use this strategy outside a lab setting, the strategy would require one to have foresight and plan for having cash on hand by visiting an ATM or bank before going shopping. In sum, strategy type may differ depending on context and the moderation results should be interpreted with caution.

## Study 2: Financial self-control strategies in the online media

It is unlikely that a lay person would turn to academic journals to learn about self-control and pursuing goals. They would most likely just “Google it”. In the next study, we conducted an analysis of the financial self-control strategies communicated in a sample of online media. This media perspective allows us to examine how many of the empirically tested self-control strategies are covered in online media communications, addressing how well the academic knowledge is mobilized and reaches the public via online media communications.

### Methods

#### Procedure

A sample of unique online webpages about financial self-control strategies was collected from Google.com (the most popular search engine worldwide, [71]) in January 2020. The search terms were specific to spending strategies: “save money”, “spend less money”, “strategies to save more money”, “strategies to spend less money”, “save more money AND self-control OR willpower”, “spend less money AND self-control OR willpower”. A pilot test with a sample of US MTurk workers asked people to give the search terms they would use if they were searching on Google for strategies that would help them save more or spend less money; of the 373 search terms provided, 78.8% (*n* = 294) included at least one keyword that matched the search terms used in Study 2. The search was restricted to English language webpages and webpages created in the last 10 years. The first 30 results for each search term were included (this is equivalent to the first three pages of a default Google search; note that only 5% of internet users continue to page two; [72]), resulting in 180 webpages. Duplicate webpages (*n* = 54) and irrelevant webpages (e.g., e-books, forums; *n* = 22) were excluded, resulting in a final sample of 104 webpages. See the OSF link for the collected webpages and media strategies.

Next, three raters coded the webpages in three steps. First, raters counted the number of self-control strategies listed on the webpage. Second, raters coded each strategy for the strategy category (e.g., create/use budgets, avoid spending temptations, automatize savings). Third, raters coded each strategy for the strategy type (i.e., proactive or reactive).

One coder (the first author of this paper) coded all 104 webpages, and two research assistants coded 52 webpages each. Thus, each webpage was coded by two independent raters (i.e., the first author and an additional rater). Overall, there was moderate to strong agreement (κ range = .70 to 87; [32]) between the raters: agreement for strategy category, κ _first author/rater1_ = .874, *SE* = .019; κ _first author/rater2_ = .805, *SE* = .022, agreement for strategy type, κ _first author/rater1_ = .769, *SE* = .020; κ _first author/rater2_ = .755, *SE* = .019, agreement for strategy outcome: κ _first author/rater1_ = .710, *SE* = .046; κ _first author/rater2_ = .799, *SE* = .044. Inconsistencies were resolved by the first author.

### Results

Across 104 webpages, the online media sample included 1,950 suggestions for how to reduce spending or increase saving. On average, there were 17.91 suggestions per webpage, ranging from 1 to 206 per webpage. Of these suggestions, 56.3% (*n* = 1098) were not self-control strategies (e.g., suggestions to downsize, earn more money, move to a cheaper city). In total, the online media sample included 852 self-control strategies from 104 webpages. About half (52.9%) of the websites specialized in financial topics (financial organizations *n* = 29, financial blogs *n* = 19, financial newspapers/magazines *n* = 7) and the other half did not (other organizations *n* = 26, other blogs *n* = 11, other newspapers/magazines *n* = 12).

Strategy categories are listed by frequency in Table 3. The three most commonly promoted strategies were being a savvy shopper (e.g., using coupons, purchasing on sale items only, shopping around for the best price), avoiding tempting situations (e.g., not browsing online/in store, avoiding shopping when hungry or in a bad mood, going to the library to rent movies and books), and avoiding spending situations by “doing it yourself” (e.g., bringing lunch to work, making your own coffee, wash your own car). Of all strategy categories identified in the online media sample, 13 (out of 22 strategy categories listed) overlapped with strategies identified in the meta-analysis of empirical papers. Put differently, 472 (55.4%) of the 852 self-control strategies listed in the online media sample were strategies for which the strategy category overlapped with one of the 18 strategies collated in the review of the academic literature.

**Table 3:** Financial self-control strategies and frequency of mention in a media sample (Study 2).

Strategy category	Count	%	Meta-analysis
Savvy shopping (e.g., use coupons, only buy on sale items, shop for best price, avoid brand name items)	181	21.2	[55]
Avoid tempting places, people, activities (e.g., restaurants, smoking, malls, online browsing)	139	16.3	
Avoid spending temptations by doing it yourself (e.g., make your own lunch/coffee, fix your own car)	108	12.7	
Make a plan (e.g., shopping lists)	57	6.7	[45]
Automatize (e.g., automatic deductions from paycheck)	55	6.4	[36]
Create or use budgets/mental accounting	55	6.4	[44, 51]
Track accounts/spending or saving behaviour	40	4.7	[35]
Make money hard to access (e.g., leave wallet at home, freeze credit/debit cards, cut up access card to saving account)	36	4.2	[41]
Set or think about your financial goals (e.g., retirement, vacation, college fund)	35	4.1	
Think about if you need or want it	31	3.6	
Pay cash only	26	3.0	[6–8]
Wait before making the purchase	25	2.9	
Rely on others for support (e.g., give husband credit card, ask girlfriend before buying, financial advisor)	14	1.6	
Think about or imagine your future self	7	0.8	[47]
Translate money into time spent working	6	0.7	
Think about or imagine your future regret	4	0.5	[54]
Choose to pay now rather than later	4	0.5	[53]
Use a retirement savings projection plan	3	0.4	[34]
Apply for a savings account with no early withdrawals	2	0.2	[38]
Save before spending (e.g., save 50% of paycheque, limit spending by saving first)	2	0.2	
Use rewards to motivate self	2	0.2	
Think about the reasons for your financial goal	2	0.2	[48]
Other strategies (strategies mentioned only once)	18	-	

Note. Strategies included in this table were mentioned at least twice, otherwise they were included in the ‘Other Strategies’ category. References in the Meta analysis column refer to academic literature on a similar strategy.

Most of the self-control strategies in the online media sample were proactive strategies (*n* = 684, 80.2%) rather than reactive strategies (*n* = 168, 19.7%). There was no significant difference between finance focused vs. other webpages and the types (proactive vs. reactive) of recommended strategies, χ^2^ (1, *N* = 851) = 0.36, *p* = .551. Finance focused webpages promoted as many proactive strategies (*n* = 55; 32.1% proactive strategies) as other webpages (*n* = 49; 39.6% proactive strategies).

### Discussion

This study showed some overlap of the kind of strategies mentioned in an online media sample with the kind of strategies studied in the academic literature, according to our meta-analysis. The most common strategy in the online media is being a savvy shopper, which includes using coupons. However, empirical evidence [55] suggests that using coupons actually *in*creased spending. Only about half of the strategies from the academic literature search were found in the online media sample, suggesting that there is still a large gap between what is studied by researchers and what is recommended online. Communications in the online media sample focused more heavily on proactive (vs. reactive) strategies than the empirical studies in the meta-analysis. Proactive strategies may be perceived as more effective by journalists/media writers and therefore be prioritized, similar to the theories posited by researchers (e.g., [5, 25]).

Note that this sample of online media was a convenience sample. Search results would change with time, keywords, and the country code of the Google site. Our aim was not to find an exhaustive list of strategies communicated in the online media, rather we aimed to collect a sample of the online discourse on financial self-control strategies.

## Study 3: Financial self-control strategies reported in the lay population

Do the strategies tested in the academic literature or present in the online discourse align with people’s daily behaviour? We asked individuals to describe the strategies they use in their daily life and then coded these self-reported strategies based on outcome and strategy type.

### Method

We recruited 1000 Canadian and American MTurk workers in August 2019. Sixty-one participants were excluded because they failed an attention check, resulting in a final sample of 939 participants (46.8% female; *M*_age_ = 37.22 years, *SD*_age_ = 11.57). Most participants (64.3%) had a college degree or higher. Participants’ median personal income before taxes was $30,000 - $39,999. See the OSF link for the verbatim responses from participants. This study received ethics approval from Carleton University Research Ethics Board–B (approval #111059).

After providing informed consent, participants reported demographic variables: age in years, gender (male, female, other), education (high school degrees or less, some college or university experience, college or trade school degrees, undergraduate degree, graduate university degree), and personal income (in binned categories from under 20,000 = 1 to over 150,000 = 11). As part of a larger questionnaire on financial decisions, participants were asked in an open-ended question, “What kinds of strategies do you use in your own life to limit spending and increase saving?”. On average, participants wrote 24 words (*SE* = 0.67) in this description. Three raters coded these descriptions for strategy category and strategy type (proactive vs. reactive). When multiple strategies were listed, only the first strategy was coded. There was moderate agreement between coders (κ range = .35 to .62; agreement for strategy category: κ _rater1/rater2_ = .59, *SE* = .02; agreement for strategy type: κ _rater1/rater2_ = .35, *SE* = .02; [32, 33]). Inconsistencies were resolved by discussion and remaining inconsistencies were resolved by a third coder (the third author).

### Results

Among the descriptions, 41 participants said they did not use any strategies and 68 participants did not describe a *self-control* strategy (e.g., instead they reported working more, investing money). The remaining 830 participants described a financial self-control strategy that was coded. Table 4 lists the strategy categories that emerged from the coding by frequency.

**Table 4:** Financial self-control strategies, examples, and frequency of mention in a lay person sample (Study 3).

Strategy category	Example (direct quotes from participants)	Count	%	Meta-analysis	Media sample
Create or use budgets/mental accounting	*Keep a strict by the penny budget*. *Make sure to include free and inexpensive small pleasures*. *Wish list for food and other items I cannot afford now*.	138	17.3	[44, 51]	✓
Think about if you need or want it	*When I want something*, *I ask myself how much I really need it or if I’ll actually use it before I decide if I’ll buy it or not*.	109	13.7		✓
Make money hard to access (e.g., leave wallet at home, freeze credit/debit cards, cut up access card to saving account)	*I have put my credit cards in a jar of water and put this in the freezer*. *It’s prevented me from impulse spending*.	95	11.9	[41]	✓
Avoid tempting places, people, activities (e.g., restaurants, smoking, malls, online browsing)	*If shopping*, *I go only to the aisles where the items I need are located*. *I don’t allow myself to window shop unnecessary things or I will buy them*.	70	8.8		✓
Savvy shopping (e.g., use coupons, only buy on sale items, shop for best price, avoid brand name items)	*I use coupons and research sell prices to maximize savings*.	49	6.2	[55]	✓
Save before spending (e.g., save 50% of paycheque, limit spending by saving first)	*I save at least 40% of what I earn each month*, *after I pay all the necessary bills in that month*.	47	5.9		✓
Set or think about your financial goals (e.g., retirement, vacation, college fund)	*My husband and I just keep talking about our goal of building a house*, *so it’s top of mind all the time to keep me from spending when I shouldn’t*.	41	5.2		✓
Track accounts/spending or saving behaviour	*I keep a chart of all of my expenses for the month and track my spending and savings over time*.	40	5.0	[35]	✓
Think about or imagine your future self	*Think of my kids future*, *think of myself where I want to be like retired and how it would feel to be still working into retirement*.	39	4.9	[47]	✓
Wait before making the purchase	*Whenever I have the desire to buy something*, *I wait a week*, *and if I still want to buy it*, *then I buy it after checking for the best deal*.	28	3.5		✓
Automatize (e.g., automatic deductions from paycheck)	*I have made a ’bill’ that automatically comes out of each paycheck that is deposited into savings*. *This way it is gone before I can spend it*.	27	3.4	[36]	✓
Rely on others for support (e.g., give husband credit card, ask girlfriend before buying, financial advisor)	*I gave my husband the password to my credit card account to keep me honest and help me stay on track with my spending*!	22	2.8		✓
Pay cash only	*I have recently started a new method of taking out $200 in cash on Sunday’s*. *It helps me to see exactly what I have spent and what I have left*.	18	2.3	[6–8]	✓
Use rewards to motivate self	*For every $1000 I save I buy a new purse for myself*.	17	2.1		✓
Think about or imagine your future regret	*I tell myself that I will regret wasting money when I desperately need it*.	16	2.0	[54]	✓
Think about your past or current debt/financial problems	*I just think about how horrible it was for me when I was completely broke–I literally had nothing*, *was sleeping in an airport*, *I had decimated credit*. *Thinking of that puts things into perspective*.	13	1.6		
Translate money into time spent working	*I try to think of the amount of time I spent making the money I am spending*.	12	1.5		✓
Make a plan (e.g., shopping lists)	*I create a shopping list for myself*. *If it’s not on the list*, *then I don’t buy it*.	9	1.1	[45]	✓
Avoid spending temptations by doing it yourself (e.g., make your own lunch/coffee, fix your own car)	*Do my own nails and color my own hair*. *Eat at home often*.	4	0.5		✓
Apply for a savings account with no early withdrawals	*Put money into funds that penalize me for early withdrawal*.	2	0.3	[38]	✓
Other strategies (strategies mentioned only once)		34	-		

Note. Strategies included in the table were mentioned at least twice, otherwise they were included in the ‘Other Strategies’ category. References in the Meta analysis column refer to academic literature on a similar strategy, checkmarks in the Media sample column refer to a similar strategy mentioned on a financial advice website.

Of the 20 strategy categories identified in the lay sample, 10 (50.0%) of these strategies reported by lay individuals mapped on to the strategies identified in the meta-analysis (e.g., making money harder to access, [44]; setting specific goals, [41]). Put differently, 443 (53.4%) of the 830 self-control strategies listed in the lay person sample were strategies for which the strategy category overlapped with one of the 18 strategies collated in the meta-analysis. Nineteen (95.0%) of the strategies described by the lay sample mapped onto the strategies identified in the online media sample. Put differently, 783 (94.3%) of the 830 participants listed strategies that were similar to one of the 22 strategies collated in the online media sample.

The majority of participants’ self-reported strategies were proactive (*n* = 556, 67.0%) rather than reactive (*n* = 274, 33.0%). We examined demographic variables as predictors of the type of strategy participants listed. In a logistic regression, we entered type of strategy (0 = reactive, 1 = proactive) as outcome variable, and age, gender (1 = female, 0 = other), education level, and personal income as predictor variables. The only significant predictor of using more proactive strategies was level of education, *B* = 0.14, 95% CI [0.02; 0.24] *B*(Exp) = 1.15, *p* = .036, such that participants with graduate level university degrees (*n* = 104) reported relatively more proactive strategies (80%) than participants with undergraduate degrees (*n* = 257, 66% proactive strategies), participants with college or trade school degrees (*n* = 171, 69% proactive strategies), participants with some college or university experience (*n* = 199, 66% proactive strategies), and participants with high school degrees or less (*n* = 93, 58% proactive strategies). We present demographic variables (age, gender, income, education) by individual strategy in a supplemental file: https://osf.io/vpwfd/?view_only=ff460390e6f040f997131d907c75b4ba.

### Discussion

This study suggests that the vast majority of people use self-control strategies in their daily life, as only 4.4% of people reported that they do not use any strategies when pursuing financial goals. Furthermore, this study again suggested a gap between the literature (as summarized in the meta-analysis) and people’s day-to-day use of self-control strategies, as only about half the self-reported strategies overlapped with strategies identified in the meta-analysis. As there was a considerably larger overlap between self-reported strategies and strategies identified in the online media sample it suggests that either lay people learn financial self-control strategies from the online media or lay authors of online media articles primarily draw on their own experiences.

Note that this sample of lay person reports was a convenience sample recruited through MTurk. Thus our findings are based on a specific subset of the population (younger, more educated, and more white; [73]) and may not generalize to other populations. Also, individual differences such as age might play a role in strategy effectiveness (e.g., older people might prefer paying by cash more than younger people). People in better financial situations use more strategies that boost willpower and fewer strategies that reduce temptations [74]. It is likely that the types of strategies used differs by person, situation, group, and this research only presents a snapshot of the strategies people use in their daily life.

## Financial self-control strategies

**Fig 4:**
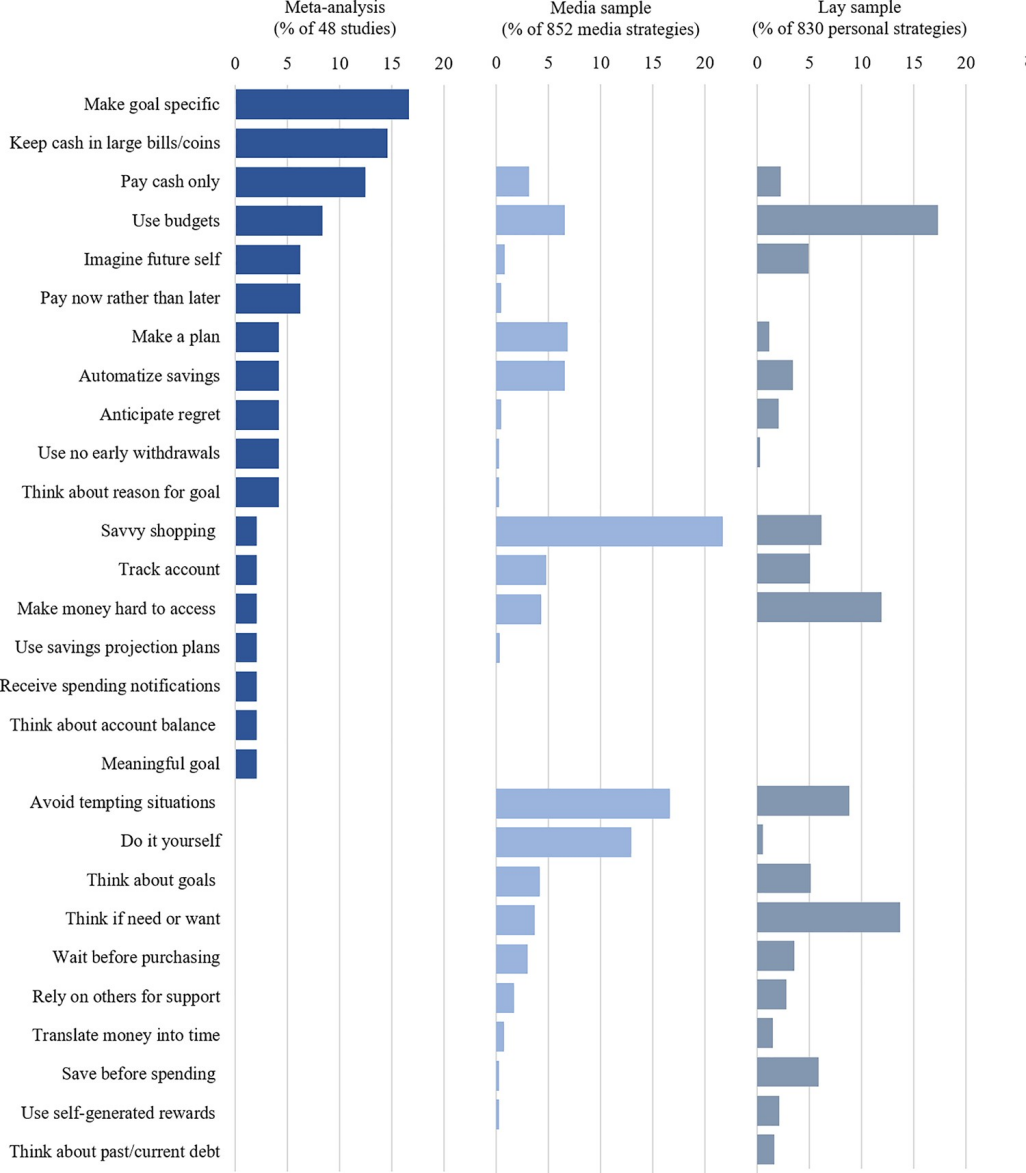
Panel graph showing prevalence of self-control strategies across the meta-analysis, media sample, and lay sample. Each panel shows the percentage of how often each strategy was mentioned by each perspective. Refer to Tables 1–3 for strategy details.

We examined the financial self-control strategies studied in the academic literature (meta-analysis), strategies recommended in a sample of online media articles on finances (Study 1), and strategies reported by a sample of American adults (Study 2). Across these different perspectives, 28 unique financial self-control strategies were identified. See Fig 4 for the relative frequency of the strategies across all three perspectives.

Approximately half of the specific strategies mentioned in an online media sample and by lay persons matched with the specific strategies identified in the meta-analysis. However, at least as many financial self-control strategies promoted in the online media and reported by lay persons have not yet been studied in the academic literature. Notably, there were seven strategies that appeared in all three perspectives: anticipating future regret [54], automatizing savings [36], imagining your future self [47], making money hard to access [41], paying only with cash [6–8], tracking accounts [35, 52], and–the most frequent across all samples–using budgets [44, 51].

The proportion of proactive relative to reactive strategies were different in the academic, online media, and lay person perspectives. As per a meta-analysis of the academic literature to date, relatively fewer strategies were proactive (proactive: 50%, reactive: 50%), compared to the online media recommendations (proactive: 80%; reactive: 20%) and self-reported strategies among lay persons (proactive: 67%; reactive: 33%).

## General discussion

Failures in self-control in the financial domain can have dire consequences. Financial security predicts overall quality of life [75] and subjective well-being [76], whereas financial stress has been linked to physical health struggles [77], problems in close relationships [78], and stress in retirement [79]. Self-control strategies can aid financial decision making, reducing spending, and increasing saving. This paper is the first attempt at collating the diverse self-control strategies that have been studied in the financial domain. A formal meta-analysis of the existing studies to date suggests that financial self-control strategies are effective in improving financial decisions (Study 1). Strategies purported in online media communications (Study 2) and those reported as part of lay persons’ financial habits (Study 3) show a considerable degree of overlap but also show that a number of financial self-control strategies that are recommended in online media and are reportedly used by lay people have not yet been studied empirically. All three perspectives showed a focus on proactive rather than reactive strategies, in line with theoretical models that argue that proactive strategies are superior to reactive strategies [5, 25]. However, proactive and reactive strategies in the meta-analysis were equally effective (Study 1) and were promoted equally by ‘financial expert’ websites or other websites (Study 2). More educated lay individuals were more likely to report proactive strategies compared to less educated individuals (strategy type did not differ age, gender, and income). It appears that both researchers and lay people with higher levels of education are drawn to self-control strategies that are used before a tempting situation occurs.

### Self-control strategies in other domains

This paper summarizes research, online media communication, and personal experiences about a relatively understudied domain of self-control: financial goals. The majority of psychological research on self-control strategies has focused on academic goals (e.g., [29, 80–84]) and health goals (e.g., [85–90]). In contrast, in the present paper we focused specifically on self-control strategies specific to the financial domain. However, these specific financial self-control strategies nonetheless link to broader conceptual types of self-control strategies such as avoiding temptations more generally (e.g., [53, 91, 92]), changing how one thinks about a temptation (e.g., [54, 93, 94]), and pre-committing before encountering a temptation (e.g., [38, 95, 96]).

Therefore, the broad findings of this research–the overlap of online media communication and lay perspectives with empirically studied financial self-control strategies and the relatively greater importance of proactive strategies among the two former perspectives–likely generalizes to self-control strategies in other domains. It might also be that self-control strategies are more effective if they are conceived with regards to the specific self-control domain. For example, the general strategy ‘avoid temptations’ may be more effective for promoting health eating when phrased as ‘avoid the dessert menu at restaurants’ and more effective for promoting reduced spending when phrased as ‘avoid certain stores in the mall’. Just as for financial self-control strategies, there may be specific self-control strategies in other domains that have yet to be tested empirically but are commonplace in daily life.

### Practical implications

A considerable portion of the existing self-control research has focused on the broader conceptualizations of self-control (e.g., [5, 10, 25]) or examined trait measures of self-control (e.g., [13, 97]). This more abstract conceptualization is useful for establishing a framework, but less useful for practical applications: It does not describe what *specific* behaviours might be enacted through self-control strategies to create meaningful change in people’s goal pursuit. In contrast, we focused on specific financial self-control strategies. Overall, we generated 28 self-control strategy categories across three perspectives (empirical studies, online media communications, lay perspectives). This list of financial self-control strategies might be fairly comprehensive as well as specific.

### Limitations

While we did not find a significant difference in effectiveness between proactive and reactive strategies in the meta-analysis, it is important to acknowledge that when these types of strategies are tested against each other directly, strategies used before encountering a temptation are more effective than strategies used in the tempting situation [29]. There are several possible explanations for the lack of a moderation in the meta-analysis. First, the strategy type might shift between experimental in-lab and field applications. The decision to pay with cash versus card might be made in the situation and may thus be reactive, but in everyday life might require someone to pick up cash before even encountering a spending situation, thus being proactive. Second, the studies included in the meta-analysis might have lacked “regulatory fit” [98] between the strategies coded as proactive vs. reactive and the specific behavioral outcomes. Greater fit between the type of strategy and the temptation might make self-control easier (see [99] for a similar argument about the benefit of fit between personality and temptation).

It is important to note that the purpose of this research was not to discover the best self-control strategy. Daily life involves many diverse spending situations, and some situations may not suit some strategies (e.g., using cash rather than credit reduces spending but cash cannot be used in online transactions). Over time, people may have tried various self-control strategies and settled on those that were effective for them and their personal situation. Even though some specific financial strategies showed stronger effect sizes in the meta-analysis (Study 1) or were more frequently mentioned in online media (Study 2) or by lay people (Study 3), we do not have enough evidence to suggest that these strategies outperformed other strategies. It may be better to inform individuals of a variety of possible strategies to choose from as the situation allows.

We also caution that the present research cannot represent an exhaustive list of all possible financial self-control strategies, or even all empirically tested financial self-control strategies. Even though we included broad search terms in our meta-analysis, the meta-analysis search may have missed relevant studies. This limitation could be addressed by including even broader search terms in a systematic literature review or taking a different approach and focusing on the self-control literature (regardless of financial outcomes) instead.

Similar, conclusions drawn from the online media sample and lay sample are necessarily limited by the inclusiveness of these samples. Both represent the perspectives of WEIRD [100] culture and might not generalize to other populations or global media. Additionally, media advice encompasses a variety of communications, from print media (magazines, newspapers, books) to social media (Twitter, Facebook). Our media sample focused on online Google search results, including blogs, online versions of magazines and newspapers, and websites for financial organizations. Arguably, online searches are an important and commonly used way to learn about an issue (e.g., Google processes 3.5 billion searches per day, [101]), but it is important to keep in mind that the online media sample is just that: a sample of the available communications about financial strategies.

### Future directions

Future research may examine which strategies are best in which situations, or even examine the optimal number of strategies to employ when making financial decisions. Indeed, simply providing people with a list of all possible self-control strategies may be mentally taxing. For example, one effective financial self-control strategy would be to pay for one’s purchases with cash [7]; however, in practice, this strategy would require a considerable amount of foresight and planning (i.e., additional self-control tasks) to ensure one always has cash on hand. Using this strategy in conjunction with other self-control strategies before every purchase, such as checking your account balance [51] and reflecting on future regret [54], may be too effortful considering the numerous spending situations individuals encounter on a daily basis. Thus, more selective use of self-control strategies depending on an individual’s personality and the specific spending situation may be most effective. There is likely no single, perfect financial self-control strategy to reduce spending or increase saving, and future research might examine whether educating people on sets of likely strategies can reduce their everyday spending. Future research could also examine how the strategies discovered in previous work on self-control strategies across goal domains [30, 31] compare to the strategies identified in the present research which focused exclusively on financial goals.

To address the limitation of sample representativeness, future research should examine a more representative sample of the American population, or examine strategy use across countries As Study 3 was relatively more educated and overrepresented young/middle aged adults their strategy use might have reflected a specific subset of the population (see online supplements for a comparison of Study 3 sample demographics with U.S. census demographics: https://osf.io/vpwfd/?view_only=ff460390e6f040f997131d907c75b4ba). Other subsets might use different strategies more frequently: for example, people closer to retirement may think about their future self more often as financial strategy than young adults further away from retirement.

Some subsets of the population may even reveal greater overlap between the academic research and the lay perspectives. For example, in a sample of undergraduate psychology students (a common convenience sample of many self-control studies) there may be greater awareness of the academic literature on self-control, and compared to other undergraduate samples, the strategies psychology undergraduates use may especially overlap with the strategies found in the meta-analysis literature search. Research on a more diverse sample could verify the results of the present research and can create a more complete list of financial self-control strategies. Sample demographic variables such as age and gender may not only moderate strategy use, but also strategy effectiveness for bringing spending and saving behaviour in line with people’s goals.

The meta-analysis showed that financial self-control strategies were effective but also showed that the vast majority of studies to date are lab-based. Future studies might examine whether these strategies as effective when used outside the lab, in lay person’s day-to-day life. Future studies might also study the effectiveness of the strategies that emerged from lay and online media perspectives but have not yet been studied empirically, in the lab or in the field.

## Conclusions

Falling short of financial goals has the potential to be disastrous for a person’s life. Exerting self-control to bring spending in line with goals can be difficult. Self-control strategies that are designed to reduce spending and increase saving have the potential to make this process easier. Three studies collected an array of financial self-control strategies sampled across three perspectives–the academic, online media, and lay-person perspectives. These perspectives overlapped to a considerable degree (e.g., using budgets was mentioned across all perspectives as strategy to decrease spending) but there was a gap between what academics study and what people do in their daily life (e.g., using coupons was mentioned as strategy to decrease spending by lay persons and in the online media sample yet increased spending according to research; recruiting other people to support financial decision making was mentioned by lay persons and in the online media sample but has not yet been studied, to our knowledge). There was greater agreement between the online media and lay perspectives than of either with the academic perspective, suggesting that individuals’ personal spending habits follow online media recommendation rather than academic findings. In sum, this paper shows that there are a multitude of strategies to support financial goals. We hope that readers of this article will find inspiration for their personal everyday financial spending from this compilation of possible strategies. We also hope that academics among the readers will find inspiration to study some of those self-control strategies recommended in the online media and used by lay individuals that have not yet been tested empirically.

## Supporting information

S1 Checklist PRISMA 2009 checklist. (DOC)
